# Lack of Short-Term Effectiveness of Rotating Treadmill Training on Turning in People with Mild-to-Moderate Parkinson's Disease and Healthy Older Adults: A Randomized, Controlled Study

**DOI:** 10.1155/2012/623985

**Published:** 2011-12-04

**Authors:** Marie E. McNeely, Gammon M. Earhart

**Affiliations:** ^1^Program in Physical Therapy, Washington University in St. Louis, St. Louis, MO 63108, USA; ^2^Program in Neuroscience, Division of Biology and Biomedical Sciences, Washington University in St. Louis, St. Louis, MO 63110, USA; ^3^Department of Anatomy and Neurobiology, Washington University in St. Louis, St. Louis, MO 63110, USA; ^4^Department of Neurology, Washington University in St. Louis, St. Louis, MO 63110, USA

## Abstract

Since turning is often impaired in Parkinson's disease (PD) and may lead to falls, it is important to develop targeted treatment strategies for turning. We determined the effects of rotating treadmill training on turning in individuals with PD. This randomized controlled study evaluated 180° in-place turns, functional turning (timed-up-and-go), and gait velocity before and after 15 minutes of rotating treadmill training or stepping in place in 26 people with PD and 27 age-matched controls. A subset of participants with PD *(n* = 3) completed five consecutive days of rotating treadmill training. Fast as possible gait velocity, timed-up-and-go time, 180° turn duration, and steps to turn 180° were impaired in PD compared to controls (*P* < 0.05) and did not improve following either intervention (*P* > 0.05). Preferred pace gait velocity and timing of yaw rotation onset of body segments (head, trunk, pelvis) during 180° turns were not different in PD (*P* > 0.05) and did not change following either intervention. No improvements in gait or turning occurred after five days of rotating treadmill training, compared to one day. The rotating treadmill is not recommended for short-term rehabilitation of impaired in-place turning in the general PD population.

## 1. Introduction

Parkinson's disease (PD) is a progressive neurodegenerative disease resulting in a variety of motor symptoms. Individuals with PD frequently experience difficulty with gait and turning, with more than half reporting difficulty turning [1–3] which may result in falls and serious injuries [4]. Symptoms of PD are treated using various therapeutic approaches; however, there are currently no effective treatment options that specifically target turning difficulty. Turning difficulties, including increased time to turn and increased number of steps to turn, are present even when individuals with PD are on PD medications [5–10]. 

Stepping in place on the rotating treadmill has been recommended as a possible rehabilitation option for those with PD [11]. After stepping in place on the rotating treadmill, healthy controls and people with PD show a rotational adaptation response known as podokinetic after-rotation [12–14]. The kinematics of podokinetic after-rotation are similar to those seen during normal in-place turning [11]. It has been suggested that the rotating treadmill may improve turns by serving as an external cue to promote the correct motor programs for successful turning [11].

Immediately after stepping in place on a rotating disk for a total of 15 minutes on one day, turning performance was improved in two people with PD on medication who also experienced freezing of gait during turns [15]. Specifically, there were fewer freezing events, reduced time to turn, less variable vastus lateralis muscle activity, and reduced coactivation of bilateral vastus lateralis muscles [15]. It remains unclear whether improvements seen with rotating treadmill training are specific to people with PD who experience freezing, nor do we know if improvements occur in other aspects of turning. If turning improvements occurred in a more diverse group of individuals with PD, the rotating treadmill would potentially be relevant as a rehabilitation tool. Our aim was to conduct a randomized controlled study examining the effects of rotating treadmill training on in-place turning in a larger, more representative group of individuals with PD and age-matched healthy controls. This study also includes control exercise groups for PD and healthy older adult participants. These control groups stepped in place on the floor for an amount of time equal to the treadmill training performed by the other groups. We hypothesized that turning would improve in individuals with PD following rotating treadmill training, while turning would likely remain unchanged for all healthy older adults and for those with PD who stepped in place on the floor.

## 2. Methods

### 2.1. Participants

We recruited 29 participants from the Movement Disorders Center at Washington University School of Medicine who had been diagnosed with idiopathic Parkinson's disease according to standard criteria [16]. We also recruited 28 older adults without PD. People with PD were recruited if they were taking medication for PD, were ambulatory, did not have deep brain stimulators implanted, had no history or symptoms of other neurological diseases, and had no recent surgeries or injuries affecting walking or turning. Those with PD were tested approximately one hour after their last dose of PD medication. Of the 29 people with PD recruited, 3 did not complete the study due to fatigue. These individuals were excluded from all subsequent analyses. Older adults without PD were recruited if they were ambulatory, had no history or symptoms of neurological diseases, and had no recent surgeries or injuries affecting walking or turning. Of the 28 controls recruited, one did not complete the study due to fatigue and was excluded. Demographics for included participants are shown in Table 1. All participants provided written informed consent prior to participation, and this study was approved by the Washington University School of Medicine Human Research Protection Office.

### 2.2. Experimental Design

All participants with and without PD completed testing on one day, and a small subset of participants with PD (*n* = 3) returned for an additional five consecutive days of training and testing. Training and testing sessions were identical for the one-day and five-day portions of the study. Surgical skin pens were used to ensure consistent placement of reflective markers across days. The Movement Disorders Society Unified Parkinson's Disease Rating Scale motor subscale (MDS-UPDRS-III) was given to all participants prior to testing to assess movement impairments [17].

### 2.3. Intervention

Participants with and without PD were randomly assigned (computer-based algorithm) to an intervention condition (rotating treadmill (Train) or stepping in place (Step)). Those in the Train condition were asked to step in place on the perimeter of a rotating disk built into the floor (120 cm diameter, Neuro Kinetics, Inc., Pittsburgh, Pa) as it rotated approximately 45°/sec either clockwise or counterclockwise. The direction of treadmill rotation was selected for each participant to train turns in the worse direction (i.e., the direction requiring greater time to turn in place 180°), where clockwise rotation trained left turns and counterclockwise rotation trained right turns. For the five-day training sessions, right turns were trained for all participants. Participants walked on the rotating treadmill for a total of 15 minutes, divided into 5-minute blocks with interspersed 5-minute rest periods [14]. Those in the Step condition experienced a similar amount of physical activity by stepping in place at a self-selected pace on the stationary ground for a total of 15 minutes, divided into 5-minute blocks with interspersed 5-minute rest periods.

### 2.4. Data Collection and Analysis

Turning and walking were assessed in two separate blocks, once before (PRE) and once after (POST) the assigned intervention. In each testing block, we examined gait, functional turning while walking, and in-place turns of 180°. Gait was assessed using a 4.8 m GAITRite instrumented walkway (CIR Systems, Havertown, Pa) to determine if the interventions had any effects on gait. The GAITRite calculated gait velocity in six walking trials in each block: three at a participant's preferred pace and three as fast as possible. Functional turning ability was assessed using the timed-up-and-go (TUG) test where participants rise from a chair, walk three meters, turn 180°, walk three meters back to the chair, and sit down. Participants completed the TUG six times in each block, with instruction to turn left or right on each trial (3 trials of each, randomized). The time to complete this task was measured using a stopwatch. 

To assess in-place turning, we instructed participants to stand in the middle of the room and turn 180° to the right or left, 10 times in each direction (randomized), to face the wall behind them. Kinematic data were recorded at 100 Hz during these turns using an eight-camera 3-D motion capture system (Motion Analysis Corp., Santa Rosa, Calif). Thirty-four reflective markers were placed on each participant: four on the head (forehead, back of the head, and above the left and right ears), seven on the trunk (left and right acromion processes, right scapula, seventh cervical vertebra, tenth thoracic vertebra, sternal notch, and xiphoid process), five on the pelvis (left and right anterior superior iliac spines, left and right posterior superior iliac spines, and sacrum), and nine on each leg (greater trochanter, anterior thigh, lateral femoral condyle, tibial tuberosity, middle tibia, lateral malleolus, calcaneus, navicular, base of the second metatarsal). 

The Motion Monitor software (Innovative Sports Training, Inc., Chicago, Ill) was used to create body segment models for the head, trunk, pelvis, and feet based on marker positions and to export segment rotation data. Custom written Matlab programs were used (MathWorks, Inc., Natick, Mass) to determine rotation onsets and offsets for the head, trunk, pelvis, and feet body segments, using a 5° yaw plane rotation threshold criterion. The onset of rotation of the foot used for the first step was designated as the turn onset, and the offset of rotation of the foot used for the last step was designated as the turn offset. To quantify body segment rotation sequences during turn initiation, we examined the timing of the onsets of the head (HTO), trunk (TTO), and pelvis (PTO) relative to turn onset. All onset times were expressed as a percentage of the first gait cycle of the turn. To assess overall turn performance, we also determined the number of steps used to complete each turn and turn duration. Kinematic, TUG, and gait velocity data were averaged across trials within each participant for the PRE block and for the POST block.

### 2.5. Statistical Analyses

Our primary variables of interest were functional turning ability (TUG), 180° in-place turn duration, and normalized rotation onset of the head, trunk, and pelvis relative to turn onset, to quantify timing of body segment rotations during turn initiation. Secondarily, we looked at number of steps to turn 180°. We also examined velocity during preferred pace and fast as possible gait to determine if the interventions, specifically the rotating treadmill, impacted gait. In the Train groups, we trained the worse turn direction, and the other turn direction was untrained. In order to similarly compare turn performance for the Step groups, we designated the worse turn direction and the better turn direction based on turn durations. For statistical comparisons across groups, the trained direction of the Train group was compared with the worse direction of the Step group, and the untrained direction of the Train group was compared with the better direction of the Step group. We were primarily interested in the trained direction, as turns in this direction were expected to be affected by rotating treadmill training. Separate RM-ANOVAs were run (RM factor: Time; between subjects factors: Condition, Group) for our primary variables of interest for the trained/worse directions. We also ran RM-ANOVAs for step number and gait velocity. Only 3 participants with PD completed the 5-day training component, so formal statistical tests were not used. However, we examined POST data from Day 1 and Day 5 for primary variables of interest, step number, and gait velocity to determine trends. Secondarily, we also evaluated variables similarly for the untrained/better directions.

## 3. Results

### 3.1. 1-Day Training

The PD and CN groups did not differ significantly in age (*P* = 0.503). The PD Train and Step groups had similar ages, MDS-UPDRS-III scores, and disease durations (*P* > 0.05). Similar ages and MDS-UPDRS-III scores were also seen across CN Train and Step groups (*P* > 0.05). There were no significant differences in any variables at baseline for turns in the trained/worse direction or turns in the untrained/better direction (*P* > 0.05). There were also no significant differences between those with left as the trained/worse turn direction and those with right as the trained/worse direction (*P* > 0.05), so data were combined for analysis.

#### 3.1.1. Gait and Functional Turning

**Figure 1:**
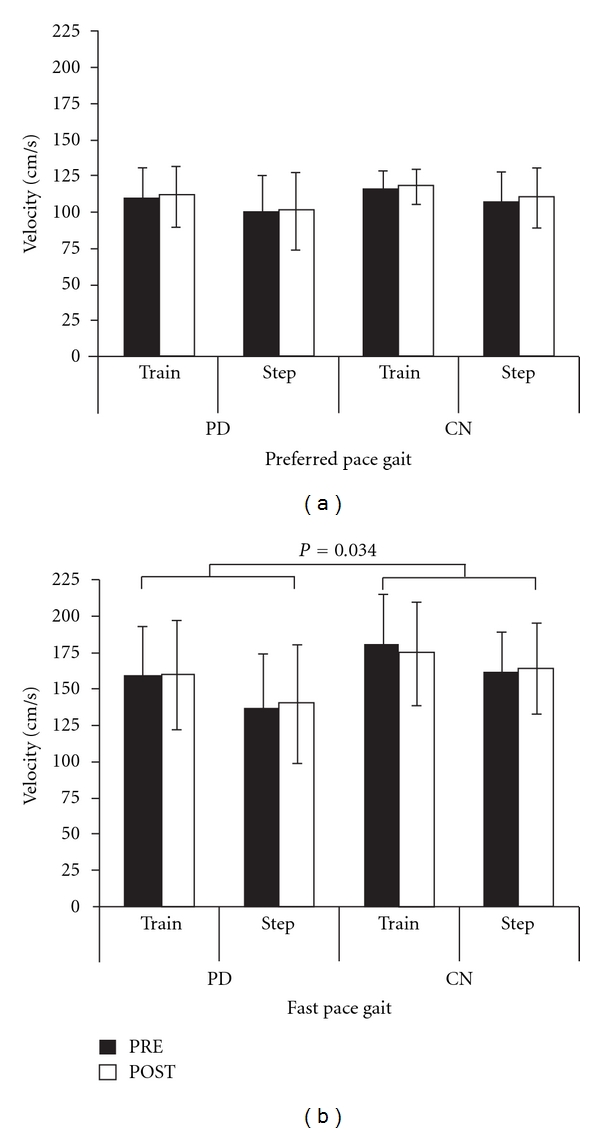
Gait Velocity. Mean preferred pace gait velocity (a) and fast as possible gait velocity (b) for PD and controls in the rotating treadmill training group (Train) and the stepping-in-place group (Step). Brackets indicate a significant group effect. Error bars are SDs.

GAITRite data for two participants (1PD, 1CN) were lost due to hard drive failure, but all remaining data for these participants was included in analyses. The mean velocity data are shown for PD and controls before and after the assigned intervention in Figure 1. There were no significant effects of Condition (f(1,47) = 2.57, *P* = 0.12) or Group (f(1,47) = 1.40, *P* = 0.24), nor any significant interaction effects (*P* > 0.05) for preferred pace gait velocity (Figure 1(a)). There was a trend towards an effect of Time (f(1,47) = 3.79, *P* = 0.06), with individuals tending to demonstrate higher preferred pace gait velocity POST intervention. For fast as possible gait velocity (Figure 1(b)), there were no significant effects of Time (f(1,47) = 0.25, *P* = 0.62) or Condition (f(1,47) = 3.28, *P* = 0.08), nor any significant interaction effects (*P* > 0.05). There was a significant effect of Group (f(1,47) = 4.77, *P* = 0.034), with PD walking slower than CN. 

**Figure 2:**
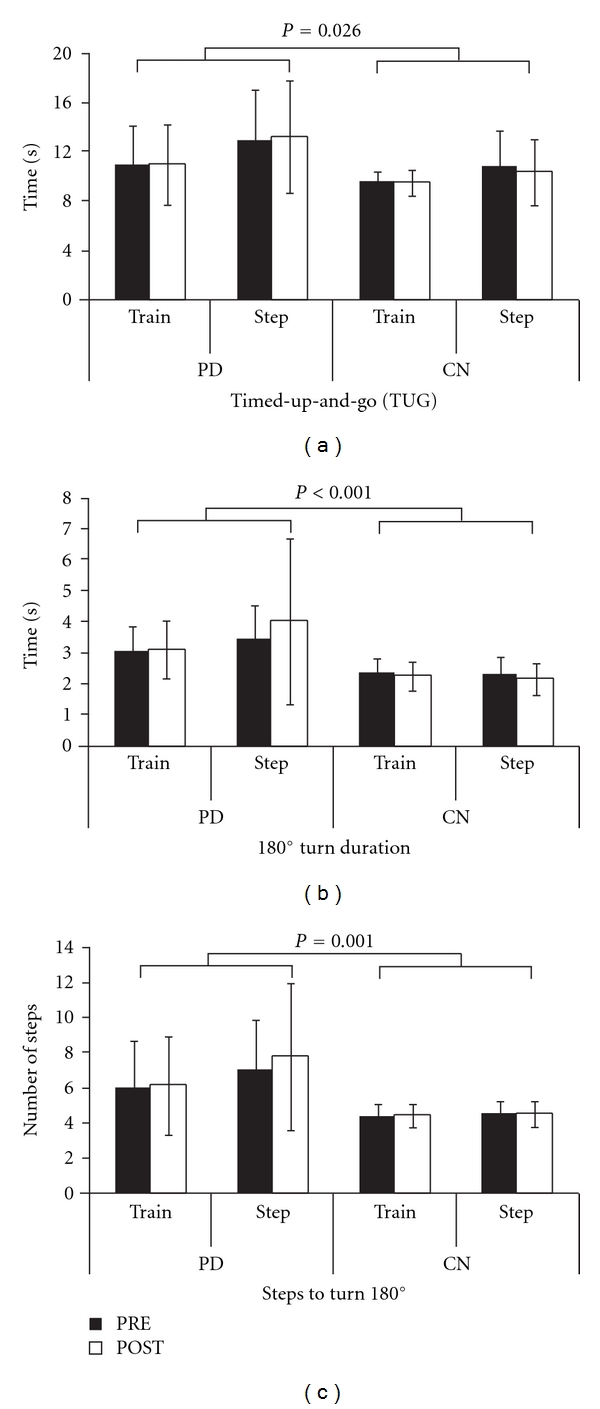
Functional Turning and Turn Performance. Mean timed-up-and-go time (a), 180° in-place turn duration (b), and number of steps to turn 180° in-place (c) for PD and controls in the rotating treadmill training group (Train) and the stepping in place group (Step). Brackets indicate significant group effects. Error bars are SDs.

For TUG where the turn component was in the trained/worse direction (Figure 2(a)), there were no significant effects of Time (f(1,49) = 0.31, *P* = 0.58) or Condition (f(1,49) = 3.43, *P* = 0.070), nor any significant interactions (*P* > 0.05). There was a significant Group effect (f(1,49) = 5.25, *P* = 0.026), with PD requiring more time to complete the TUG, turning to the trained/worse direction, compared to controls. Results were similar for the untrained/better direction.

#### 3.1.2. Turn Kinematics

For 180° turn duration in the trained/worse direction (Figure 2(b)), there were no significant effects of Time (f(1,49) = 0.025, *P* = 0.62) or Condition (f(1,49) = 0.99, *P* = 0.33), nor any significant interactions (*P* > 0.05). There was a significant Group effect (f(1,49) = 15.95, *P* < 0.001), with PD turning slower than CN. For the untrained/better direction, results were similar. 

For steps to turn 180° in the trained/worse direction (Figure 2(c)), there were no significant effects of Time (f(1,49) = 2.15, *P* = 0.15) or Condition (f(1,49) = 1.33, *P* = 0.25), nor any significant interactions (*P* > 0.05). There was a significant Group effect (f(1,49) = 13.71, *P* = 0.001), with PD requiring more steps to turn. Similar results were seen for the untrained/better direction. 

**Figure 3:**
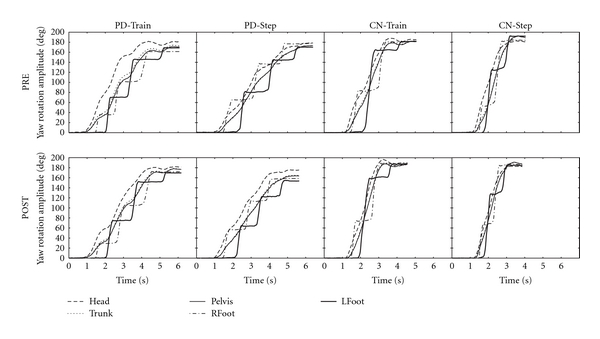
Turn Kinematics Examples. Representative traces of yaw plane rotations of individual body segments during a single 180° in-place turn in one PD-Train, one PD-Step, one CN-Train, and one CN-Step participant before (PRE) and after (POST) intervention.

**Figure 4:**
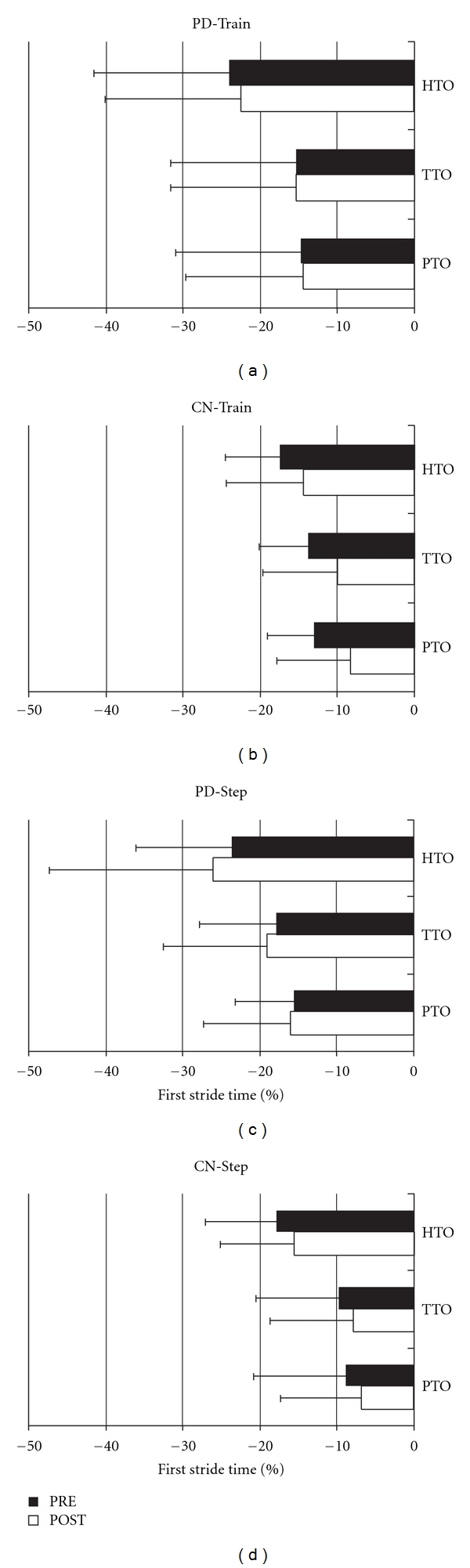
Onsets of Body Segment Rotations. Mean yaw rotation onset times of the head (HTO), trunk (TTO), and pelvis (PTO) relative to the turn onset (i.e., first foot rotation) are expressed as a percentage of the first stride of the turn for PD-Train (a), CN-Train (b), PD-Step (c), and CN-Step (d). Error bars are SDs.

For body segment (head, trunk, pelvis) rotation onsets relative to turn onset for turns in the trained/worse direction, there were no significant effects of Time (f(3,47) = 1.26, *P* = 0.30), Condition (f(3,47) = 0.60, *P* = 0.62), or Group (f(3,47) = 1.48, *P* = 0.23), nor any significant interactions (*P* > 0.05).  Figure 3 shows representative sample traces from single individuals in the PD-Train (a), CN-Train (b), PD-Step (c), and CN-Step (d) groups. Mean body segment rotation onsets are shown in Figure 4 for the PD and CN groups for the Train (a, b) and Step (c, d) conditions before and after intervention. In all groups, the sequence of rotation onsets of body segments was head first, followed by trunk, and then pelvis. Comparisons for body segment rotation onsets relative to turn onset were similar for the untrained/better direction. 

### 3.2. 5-Day Training

A small subset of the original group of participants returned for 5 consecutive days of rotating treadmill training. All three individuals who returned for five consecutive days of training were able to tolerate the training program. On average, gait velocity, TUG, turn duration, steps to turn, and body segment rotation onsets relative to turn onset were very similar following a single session of training (Day 1 POST), compared to after five sessions of training (Day 5 POST) in either the trained or untrained direction.  Table 2 shows baseline data from Day 1 prior to training, as well as from Day 1 and Day 5 after training for the trained direction.

## 4. Discussion

Difficulty with turning is common in individuals with PD, and the development of therapeutic approaches that target turn deficits might reduce the occurrence of falls and serious injuries in these individuals. As a result, it is important to evaluate potential treatment strategies in individuals with PD who demonstrate a range of turning ability and might benefit from these treatment options.

Contrary to our initial hypotheses, we did not see improvements in turning in those with PD following one day or five consecutive days of rotating treadmill training. For most turning variables, group effects indicated turning was impaired in those with PD ON medication, compared to controls, as has been previously reported [5–10]. 

Interestingly, the body segment rotation onset patterns we observed were similar between those with PD and controls. All groups initiated turns with the head, followed by the trunk, pelvis, and foot. Controls display this top-down rotation sequence during turns while walking [8, 18–24], as well as in-place turns [25]. In contrast, those with PD have been reported to display more simultaneous rotation of the head, trunk, and pelvis during turning while walking, including a pronounced delay in initiation of head rotation [5, 8, 10]. We did not see this kinematic pattern during in-place turns in people with PD. A previous report of impaired body segment rotation patterns in PD during in-place turns indicated there were no significant differences in rotation onset time between body segments [25], though average onset times of each segment followed a top-down sequence, similar to results of the present study. 

Turning and gait velocity did not systematically worsen in any intervention group from PRE to POST. Also, participants were permitted to rest as often as necessary during testing and had mandatory five-minute rests between the five-minute blocks of treadmill training. As a result, we think our results were likely not confounded by fatigue. Further, we likely tested a higher-functioning group of people with PD than some prior studies, since preferred pace gait velocity did not differ between PD and controls. Despite smaller differences in gait performance, we detected distinct impairments in turn performance compared to controls, confirming turning impairments can be present in those with relatively normal gait [5]. This highlights the need for rehabilitation to address turning deficits even in the early stages of disease progression.

The most notable difference between the present study and the previous rotating treadmill study [15] is the focus of the earlier study on individuals with severe freezing. People with PD with freezing of gait can improve motor performance via external cues, and cued exercise interventions have been used to improve locomotion in PD with freezing of gait. In one study, robot-assisted gait training improved freezing of gait frequency, as well as gait velocity, stride length, coordination, and rhythmicity in those with PD with freezing of gait [26]. It is possible that for individuals with more severe PD or with severe freezing, training on the rotating treadmill may help make the appropriate turning motor patterns more automatic. This might in turn facilitate their impaired task switching [27–29], reducing the frequency and severity freezing during turns. The present study only included 9 individuals with freezing of gait, as defined by reports of freezing at least once per week on item three of the Freezing of Gait Questionnaire [30]; however, only one individual with freezing was randomly assigned to the rotating treadmill training group, so comparisons between those with and without freezing could not be made. The small overall sample size and the fact that only one person with freezing trained on the rotating treadmill are limitations of the study and warrant careful interpretation of the data, as the study may have been underpowered to detect interaction effects.

Another limitation of the study is that the sessions were of relatively low intensity and were few in number. It may be that more intense rotating treadmill training sessions or increased number of sessions may result in detectable changes, as previous traditional treadmill studies report improvements after completion of 10–28 training sessions of 20–30 minutes each [26, 31]. However, there are also reports of acute effects on gait from just one session of traditional treadmill training [32–34]. Another possibility is that the rotating treadmill may be more useful for people with PD when combined with other cueing strategies. Combining traditional treadmill training with auditory and visual cues improved gait speed, maximum distance walked in six minutes, and score on the Freezing of Gait Questionnaire in one study of people with PD [31].

## 5. Conclusions

Fifteen minutes of rotating treadmill training alone on one day or for five consecutive days did not affect turn performance in PD. As a result, this type of training is unlikely to serve as an effective short-term rehabilitation strategy for many individuals with PD. However, future studies should determine whether rotating treadmill training may improve turning impairments with longer training paradigms or when combined with other external cues, as well as assess its effects on performance of turns while walking in addition to the in-place turning studied here. 

## Figures and Tables

**Table 1:** Participant demographics.

	CN	CN	PD	PD	PD
	1-day train	1-day step	1-day train	1-day step	5-day train
Total *n *	14	13	12	14	3
Age (yrs)	65.3 ± 11.3	70.1 ± 11.4	69.1 ± 9.7	70.0 ± 11.2	69.0 ± 17.0
Males/females	9/5	5/8	8/4	8/6	2/1
Disease duration (yrs)	NA	NA	8.5 ± 4.9	6.6 ± 5.5	8.7 ± 5.7
UPDRS-III	NA	NA	25.8 ± 10.3	26.9 ± 7.4	27.7 ± 20.6
H & Y stage	NA	NA	2.1 ± 0.4	2.1 ± 0.7	2.0 ± 0.9

Values are means ± SDs.

**Table 2:** Five-day rotating treadmill training results for the trained direction.

	Day 1 PRE	Day 1 POST	Day 5 POST
Fwd gait velocity (cm/sec)	105.1 ± 24.1	109.8 ± 18.3	110.4 ± 24.6
Fast gait velocity (cm/sec)	149.3 ± 25.5	149.5 ± 28.0	152.5 ± 26.2
TUG (sec)	12.0 ± 3.3	12.0 ± 3.5	12.1 ± 2.7
Turn duration (sec)	2.7 ± 0.8	2.6 ± 0.9	2.5 ± 0.8
Steps to turn	4.6 ± 0.5	4.6 ± 0.6	4.4 ± 0.5
NHTO (% Gait Cycle)	−30.7 ± 11.4	−24.3 ± 7.6	−23.5 ± 12.9
NTTO (% Gait Cycle)	−25.1 ± 9.5	−24.7 ± 8.4	−22.5 ± 13.7
NPTO (% Gait Cycle)	−24.0 ± 10.7	−22.8 ± 8.8	−20.1 ± 13.1

Values are means ± SDs.
